# MIDLIFE FACTORS, GENDER, AND MIGRATION ARE KEY PREDICTORS OF COGNITIVE HEALTH IN LATE LIFE

**DOI:** 10.1093/geroni/igz038.253

**Published:** 2019-11-08

**Authors:** Sunshine Rote, Jaqueline L Angel

**Affiliations:** 1 UNIVERSITY OF LOUISVILLE, LOUISVILLE, Kentucky, United States; 2 University of Texas at Austin, Austin, Texas, United States

## Abstract

Older Latinos are at 1.5 times greater risk for Alzheimer’s disease and other dementias than non-Latino Whites (Wu et al., 2018) and there is evidence of high levels of cognitive impairment, dementia, and dementia-related neuropsychiatric symptoms among older Mexican Americans in particular (Rote et al., 2015). We use data from the Hispanic Established Population for the Epidemiologic Study of the Elderly (HEPESE, 1993/94- 2010/11, N=2,665), a national study of Mexican American 65 years and older residing in the southwestern U.S. Older adults who were in the paid labor force, except for those who worked in the agricultural sector, exhibit lower risk for cognitive impairment. The results hold for both women and men. For family size, number of children is associated with greater risk for cognitive impairment and this is especially evident among women and immigrant men. Midlife factors, gender, and migration are key predictors of cognitive health in late life.

